# First demonstration of the neuroimmune link in humans using IV Endotoxin and Intradermal Capsaicin in the face and arm

**DOI:** 10.1186/1129-2377-1-S14-P221

**Published:** 2013-02-21

**Authors:** PE Rolan, MR Hutchinson, M Buijs, J Tuke, YH Kwok, M Gentgall, D Williams, N Nillsen

**Affiliations:** 1 University of Adelaide, Australia; 2 University of Leiden, Netherlands; 3 University of South Australia, Australia

## Introduction

Animal studies convincingly show that glial activation through immune stimulation has an important role in pain generation and maintenance[1]. However demonstration of whether this pathway is relevant in humans is lacking.

## Objectives

1) to determine whether low-dose intravenous endotoxin in healthy volunteers alters baseline pain sensitivity 2) to determine whether low-dose intravenous endotoxin in healthy volunteers enhances the response to intra-dermal capsaicin in the forehead and forearm

## Methods

Study 1. 9 healthy volunteers (6M) received 0.2 ng/kg intravenous endotoxin or saline placebo in a two-way crossover study with assessments of cutaneous hyperalgesia (von Frey hairs) and allodynia (brush) as well as thermal pain thresholds and cold pain test. Study 2. 12 healthy male volunteers received 0.4 ng/kg intravenous endotoxin or saline on placebo in a two-way crossover study and received 50μg id capsaicin at 2 or 3.5 hours with assessments of flare, spontaneous pain, areas of hyperalgesia and allodynia for 1 hour post injection. Core body temperature was assessed by Vitalsense capsule, standard haematology performed and circulating cytokines (TNFα, IL-1β,6,10 measured. Peak and area under the curve (AUC) were analysed by mixed effects modelling (study 2).

## Results

In study 1, despite a rise in core temperature and changes in circulating neutrophils and lymphocytes, there was no change in pain sensitivity except a reduction in cold pain tolerance following endotoxin (p=0.045). In study 2, endotoxin enhanced the peak and AUC of allodynia (p=.005 and .02), hyperalgesia (p= .04 and .05) and flare (p=.0005 and .001) and AUC of pain (p=.05) at the forehead and enhanced peak and AUC flare in the forearm (p=.03 and.04).

## Conclusion

Low dose intravenous endotoxin, a TLR4 receptor agonist on immune cells enhances the objective and subjective response to a neuropathic-like stimulus with greater sensitivity on the face compared to the arm. This model is suitable for screening for new immune directed analgesics, including for headache.
